# Aquaculture-capture fisheries nexus under Covid-19: impacts, diversity, and social-ecological resilience

**DOI:** 10.1007/s40152-021-00213-6

**Published:** 2021-01-16

**Authors:** Aisa O. Manlosa, Anna-Katharina Hornidge, Achim Schlüter

**Affiliations:** 1 Social Sciences Department, Leibniz Centre for Tropical Marine Research (ZMT), Bremen, Germany; 2 German Development Institute/Deutsches Institut für Entwicklungspolitik (DIE), Bonn, Germany; 3 Institute of Political Sciences and Sociology, University of Bonn, Bonn, Germany; 4 Department of Business and Economics, Jacobs University, Bremen, Germany

**Keywords:** Aquaculture, Fisheries, Livelihoods, Covid-19 impacts, Resilience, Social-ecological system, Sustainability

## Abstract

The Covid-19 pandemic is a global shock that is significantly affecting coastal social-ecological systems (SES) in different parts of the world. Its widespread impacts have unravelled vulnerabilities in many aspects of society, including food systems. Our study investigated the impacts of a lockdown associated with the pandemic in the province of Bulacan, in the region of Central Luzon, Philippines, where aquaculture and capture fisheries are important and interconnected sectors. In particular, we focused on impacts related to production and market. We considered people’s coping strategies and the factors that enabled such strategies. Our investigation adopted a case study approach and drew on qualitative data analysed through thematic analysis. The findings revealed differentiated mechanisms through which aquaculture and capture fisheries production were impacted. Both were strongly affected by market disruptions but through slightly different ways. In effect, the lockdown provided the impetus for the uptake and spreading of practices that were previously peripheral, particularly in relation to market exchanges. The study also identified a variety of coping strategies, as well as the importance of social support in the form of food aid, financial assistance, and institutional livelihood assistance. Finally, it discusses the importance of diversity in food sources, the role of local food systems, and governance implications for foregrounding social-ecological resilience in short-term response and long-term recovery.

## Introduction

Aquaculture and capture fisheries are important sources of food and income for a significant fraction of the global population (FAO 2020a). Due to sustained increases in aquaculture production over the years and the relatively unchanged production levels for capture fisheries, aquaculture has been perceived to play a significant role in meeting increased demand for fish protein (Duarte et al. 2009; Hall et al. 2011). The ongoing Covid-19 pandemic is a global systemic shock with wide-ranging impacts (HLPE 2020; Naidoo and Fisher 2020). It has acutely unravelled weaknesses and vulnerabilities in multiple dimensions of societal systems, crucially including the production, marketing, and distribution of food (CGIAR 2020). Aquaculture and capture fisheries are critical food production systems that have been significantly impacted (FAO 2020b). The crisis requires not only to understand how aquaculture and fisheries are differentially impacted but, importantly, to adopt a systemic perspective and learn about how co-existent aquaculture and fisheries contribute to a coastal social-ecological system’s (SES) capacity to cope and recover from shocks.

Belton and Thilsted (2014) consider aquaculture and capture fisheries as playing complementary roles for achieving food security and nutrition. This complementarity derives from the distinct benefits that each provides, with particularly important implications for the poor. They found that while aquaculture increases fish^1^ availability and lowers fish prices, capture fisheries provides access to diverse and nutritious fish. This complementarity must be kept in mind in view of increased competition for coastal resources, including between capture fisheries and aquaculture (Belton et al. 2014; Bavinck et al. 2018). In Bangladesh, Belton et al. (2014) found that while aquaculture plays an important role in responding to demand as fisheries production stagnates, the former tends to benefit the relatively affluent more than the poor. And yet, the contributions of aquaculture and fisheries to human well-being including through food security and nutrition and poverty alleviation are often analysed separately. Belton et al. (2014) therefore call attention to the need to develop policies that address the nexus of aquaculture and fisheries. In effect, they call for nuance and a pro-poor perspective in understanding the contribution of aquaculture to food security and nutrition while emphasizing the enduring importance of small-scale fisheries.

This aquaculture-fisheries nexus needs to be considered in view of the Covid-19 pandemic because of its potential contribution to the resilience of coastal SES and related aquatic food systems (Troell et al. 2014). Resilience is a feature that allows a system to reorganize while undergoing change in order to maintain its functions and dynamics in the face of a shock or perturbation. It is constituted by adaptability of the system and a capacity to transform when present conditions become untenable (Folke et al. 2010). Adaptability pertains to the capacity to learn and to adjust responses to new conditions (Berkes et al. 2003), while transformability refers to the capacity to actively or by force create a fundamentally new system (Walker et al. 2004). The pandemic and the disruptions it has caused beyond the health sector have resulted in calls for a stronger focus on resilience, including in aquatic food systems (e.g. Béné 2020; CGIAR 2020; Harris et al. 2020; HLPE 2020; Love et al. 2020; Zimmerer and de Haan 2020) particularly in long-term recovery efforts. Calls for more resilient food systems characterised by equity and embeddedness in communities, democratic and inclusive governance, and suitability with natural ecological processes have been issued by scholars and activists long before the pandemic struck (e.g. FAO and WHO 2016; Schipanski et al. 2016). Under pandemic and lockdown conditions, understanding of effects and responses in local aquatic food systems can be further deepened by examining the characteristics and mechanisms that result in vulnerabilities and resilience.

Coping strategies, which also contribute to resilience, are ways in which individuals or groups minimise the negative effects of shocks or stressors (Nelson et al. 2007). Such strategies have been vital in enabling people to protect and maintain a level of well-being, for instance, in the form of finding ways to access food, under severely constrained conditions caused by the Covid-19 lockdowns (e.g. IPES-Food 2020; Eriksson et al. 2020). In our case study, we investigate how fish farmers, fishers, and their households coped, and identify some of the factors that enabled these. We underscore how the necessity of coping accelerates the adoption of new or hitherto marginal practices which are likely to retain importance post-Covid. We further highlight how the local scale of the food system and governance of aquatic food production in the case study facilitated these coping strategies.

This case study focuses on a coastal social-ecological system in the region of Central Luzon, Philippines. Central Luzon is among the most important regions for aquaculture production in the country while also supporting capture fisheries (Bureau of Fisheries and Aquatic Resources National Office 2017). The first Covid-19 case in the Philippines was confirmed in the latter half of January 2020 (WHO Philippines 2020). Two months later, in mid-March 2020, the government imposed a strict lockdown. Across different parts of the country, the lockdowns differed in intensity, depending on the number of confirmed Covid-19 cases in the area. The study area which is about 50 km northwest of the capital Metro Manila, the country’s epicentre of the Covid-19 outbreak, was placed under strict lockdown called enhanced community quarantine (ECQ) for 2 months. The study considers the smallholder aquaculture and fisheries nexus in the context of this pandemic. In particular, it investigates how the lockdown has impacted smallholder aquaculture and capture fisheries in the areas of production and marketing, paying attention to the divergence and convergence of experiences of fish farmers and fishers and what these imply. Evidencing the impacts in these primary pathways will not only inform short-term responses but can help frame long-term strategies in similar settings for strengthening coastal SES resilience against future pandemics and other shocks. Beyond providing empirical evidence, the place-based case study approach enabled us to consider the broader coastal social-ecological system within which the aquaculture-capture fisheries nexus is embedded. This is useful for distilling not only how the nexus shaped pandemic impacts and identifying which actions are urgently needed but also for generating insights around the role of scale of food systems and governance attributes in enabling or impeding resilience.

## Methodology

### Study area

This study adopts a qualitative case study approach. The case study focuses on three adjacent municipalities called Paombong, Hagonoy, and Malolos in Central Luzon. These municipalities are bounded to the south by Manila Bay and are traversed by rivers and estuaries which are important fishing grounds.

The most recent data for the period 2015–2017 indicated that aquaculture production in the region was among the highest in the Philippines in terms of volume and economic value (Bureau of Fisheries and Aquatic Resources National Office 2017). Most aquaculture production is done in brackishwater fish ponds in small and large scales, in a spectrum of non-intensive (i.e. organic feed such as algae), semi-intensive (i.e. combination of organic feed and formulated feed), and intensive system (i.e. formulated feed). In some parts, aquaculture production is done in fish cages and fish pens. The aquaculture commodities produced in the study area include high-value tiger prawn *(Penaeus monodon)*, milkfish (*Chanos chanos)*, various species of tilapia, shrimps, and mudcrabs. While all three municipalities are important aquaculture production areas, there are slight differences in key activities. Specifically, Paombong is a key area for fingerlings production. Most large-scale and intensive aquaculture production is operated in Hagonoy. It is also where various fish markets are located, several of which specialise in aquaculture commodities. Malolos also functions as a hub for fish trade. Water pollution from domestic and industrial sources, and particularly, from unregulated and excessive formulated feed application coupled with unregulated water disposal to natural waterways and the sea, is posing a threat to aquaculture and capture fisheries. Among fish farmers, increasing incidence of fish kills and low harvest have been have been reported to lead to lost earnings. Reduction in fish catch due to stock depletion and water pollution has led fishers to use increasingly diverse types of fishing gears. The Bureau of Fisheries and Aquatic Resources in Central Luzon (2017) estimated an average annual catch of 650 kg per fisher.^2^

Harvested and caught fish are typically traded in fish markets where consignacions are the hub of the dominant exchange structure. Consignacions^3^ purchase fish from fish farmers and fishers and sell these to vendors, traders, and exporters on consignment. They provide a risk redistribution mechanism and access to credit within the fabric of patron-client relationships. BFAR and the local government units of the municipalities are the primary state actors in fisheries and aquaculture. They are strongly networked with local fish farmers and fishers through numerous fisherfolk organizations.

### Data collection and analysis

The qualitative data used in the analysis was drawn from two data collection phases. The first phase involved intensive field work prior to the Covid-19 crisis from November 2019 to the beginning of March 2020. This phase included in-depth interviews, participant observations (e.g. fishing activities, aquaculture activities, fish markets, relevant meetings), and thematic analysis of policy and regulatory documents (e.g. national fishery law, municipal fisheries ordinances). These activities were undertaken to build contextual understanding of aquaculture and capture fisheries in the coastal social-ecological system, including how they have changed, and the role of plural institutions. A total of 67 interviews were conducted with diverse actors including fish farmers, fishers, market actors, and government and non-government actors. Initially, these were undertaken under the framework of an institutional change research. In response to the Covid-19 crisis and the urgent need to capture how fish farmers and fishers have been impacted, a second and significantly smaller phase of data collection was conducted using phone interviews. This involved follow-up in-depth interviews with a subset of 15 individuals from the first data collection phase. The questions during the phone interviews focused on the impacts of the Covid-19 crisis, how fish farmers and fishers coped, and new practices that were emerging in consequence. Data from the first and second data collection phases were analysed through a thematic analysis. This involved coding qualitative data and capturing emergent themes. In particular, the contextual background of the social-ecological system provided in the methods section drew on the first data collection phase. The findings were primarily based on analysis of the second data collection phase. The determination of production and market impacts as the areas of focus was inductive and based on the preponderance of these themes in interview responses.

## Findings

In this section, we first provide a brief overview of the Philippine government’s response to the Covid-19 pandemic, particularly the containment measures it imposed to stem the spread of Covid-19. We then present impacts on aquaculture and capture fisheries in the areas of production and market. These are followed by the coping strategies undertaken, the factors that enabled these, and in particular, the role of social and government assistance in enabling individuals and households to cope.

### Philippine government’s Covid-19 containment strategy

**Fig. 1:**
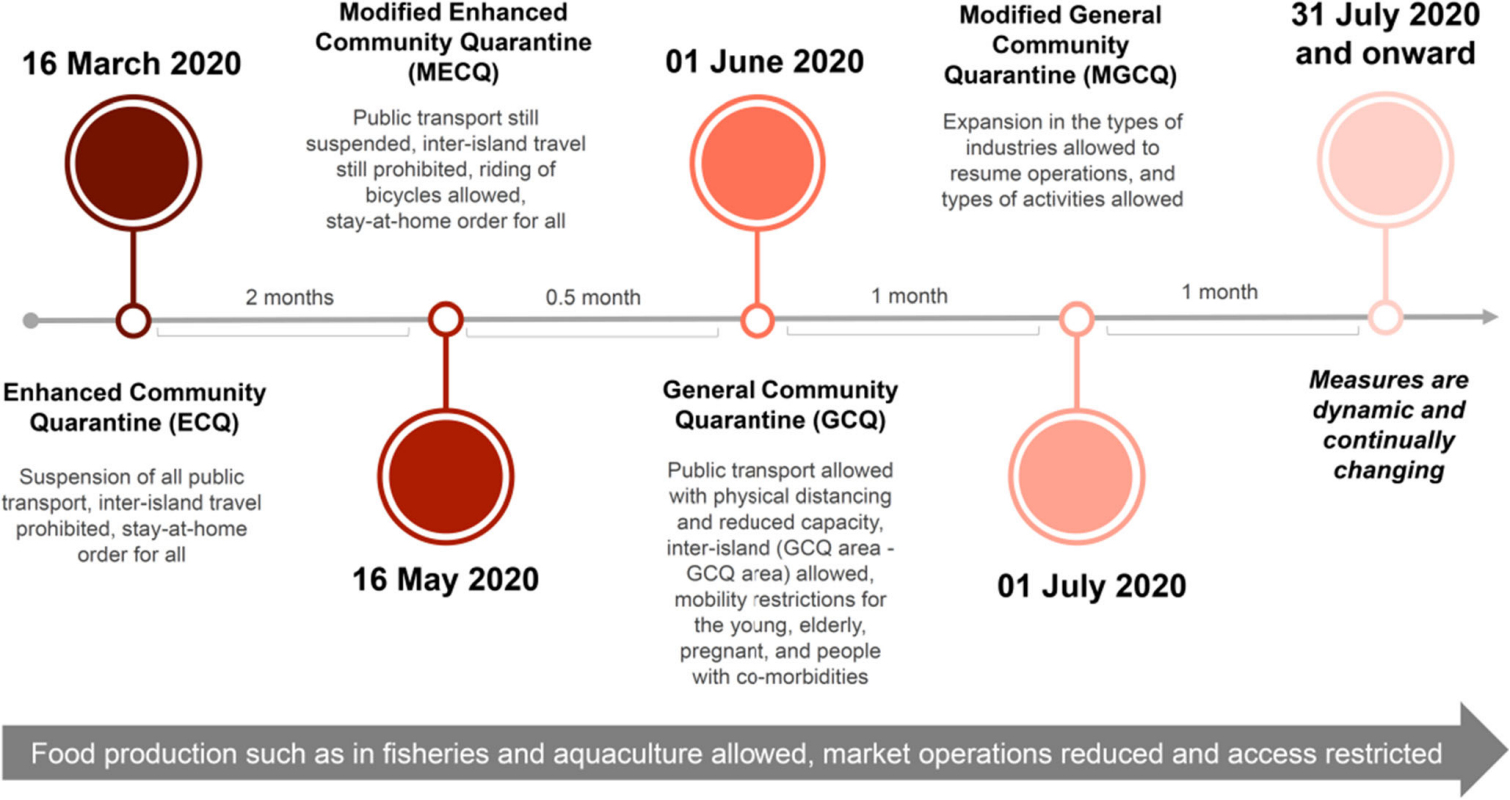
Lockdown levels from March to July 2020

Similar to other countries in different parts of the world, the Philippines implemented a set of measures to address the threat of Covid-19 in mid-March 2020 (Fig. 1). In areas with high Covid-19 cases (e.g. Metro Manila), it adopted a strict lockdown (called enhanced community quarantine or ECQ) which comprehensively restricted the movement of people and goods, exempting those that were considered essential such as the movement of frontline workers (e.g. workers in the medical system, law enforcement) and movements related to the production, distribution, and accessing of food and medicines, among others. Despite then low case counts, the study area was placed under ECQ due to its proximity to areas with a high number of cases. After 2 months in ECQ, restrictions were gradually eased despite rising daily cases due to economic considerations. Interviewees viewed the ECQ period as the most challenging and disruptive phase thus far. The impacts presented below therefore focus on this period but also include subsequent changes as restrictions were eased.

A national Inter-Agency Task Force (IATF) on Covid-19 coordinates the country’s overall response to the pandemic. Due to the devolved nature of Philippine governance, local government units at the municipality level are primarily responsible for the implementation of containment measures as well as coordinating relief and assistance efforts. During the ECQ, this resulted in the closing of borders between different municipalities which are relatively small jurisdictions. It disrupted livelihoods including that of food producers such as fish farmers and fishers. Movement restrictions between municipal boundaries were enforced by military and police who required the presentation of different types of passes to facilitate conditional mobility. For instance, quarantine passes were issued to representatives of households to allow minimal movement for accessing food and medicines. The Bureau of Fisheries and Aquatic Resources and local government units also issued a limited number of vendors’ passes to allow fish producers and market actors to move within a limited area.

### Impacts on production processes

#### Aquaculture production

Aquaculture production was negatively affected by the pandemic primarily through disrupted availability of fry and fingerlings as air travel was suspended, temporary rise in cost of fingerlings, limited local supply of various inputs due to mobility restrictions, timing of lockdown, and restricted individual mobility which reduced fish farmers’ ability to access needed inputs.

Interviewees reported that the lockdown coincided with the driest months of the year when there is typically less freshwater from the upland and fish kills are more common. Smallholder fish farmers reported fish kills either in their own fish ponds or the fish pond of someone they know. Prior to the Covid-19 pandemic, fish kills have compromised livelihoods, reduced incomes, and affected abilities to access food. However, the pandemic exacerbated the effect of this existing stressor by triggering a cascade of impacts that collectively made it challenging for smallholder fish farmers to cope. Following the fish kills, fish farmers were less able to move to access what they need. It also meant they had to access fingerlings for restocking at a time when fingerlings availability were low and the costs were high due to higher transport costs of imported fry. Others who were not affected by fish kills reported either low harvest or small fish sizes which also translated to lower income. This did not only mean less cash to purchase food and other household needs but also less capacity to invest in aquaculture production. For instance, one fish farmer reported not being able to afford to replace a damaged water gate in his fish pond. Without the water gate which enables control of the inflow and outflow of water, he had been unable to restock the pond.

Cost of formulated feeds remained relatively stable and organic feeds grown in fish ponds such as algae remained locally available. However, due to mobility restrictions, input suppliers within the study area also faced difficulties in obtaining sufficient input materials from outside the study area. “We were able to solve aquaculture inputs within our municipality but it was not enough. Those who were selling also could not easily go out. The lockdown had an effect on raising fish. It is not easy to buy feeds. When fish lack food, they go hungry which affects their size.” (Fish farmer, Paombong). Within the study area itself, fish farmers were constrained in accessing inputs such as fingerlings and feeds particularly when it involved crossing a municipal boundary due to pass requirements and mandatory quarantine of 14 days when one returns to his/her municipality, even if the distance travelled is only a few kilometres. “It was hard to get fingerlings because of the checkpoints. The price stayed the same but for the fingerlings from Bicol (a different province in the Philippines) which requires domestic air travel, the price went from Php 6 to Php 11 ($0.12-$0.22).” (Fish farmer, Paombong). Fish pond labour which is often locally sourced also remained available, and labourers were able to carry on with production activities. “We know our labourers anyway and they don’t go anywhere far so we do the same things, without social distancing.” (Fish farmer, Hagonoy). With the easing of restrictions after the first 2 months of lockdown, fish farmers have been better able to access fingerlings, feeds, and other production inputs. However, transport services continued to operate at reduced capacity and, at higher cost which in effect, still limited people’s ability to get around.

#### Capture fisheries production

During the ECQ, small fishing boats that carried only one fisher were allowed. Fishers in these boats used various types of fishing gears. They fished in rivers, estuaries, and parts of the sea near the coast. The use of fish traps and *pangangapa* (catching by hand) was particularly important for sourcing food. Larger fishing boats shared by 2 people and above were prohibited during the ECQ in order to meet physical distancing requirements. In particular, *palakaya* or commercial fishing with boats that carry 10 people or more suspended their activities. Small-scale fisheries therefore played an indispensable role in meeting subsistence needs of their own households and in supplying fish to people in their communities who were otherwise less able to access fish from public markets due to mobility restrictions. A fisher shared about the disruptions saying “It’s as if livelihoods also went on lockdown. If there is a lot of catch, then they’re used for consumption. There is rice anyway.” (Fisher, Malolos).

Similar to aquaculture as discussed above, environmental conditions also influenced the difficulties posed by the Covid-19 crisis to fishers and their households. The easing of restrictions with the transition to a modified ECQ,^4^ and later on to an even less restrictive general community quarantine (GCQ), coincided with the onset of the southwest monsoon characterised by high humidity, heavy rainfall, and low fish catch from June to October (with some variations in timing). Typically during this period, there are also less shrimps and crabs which affect those who rely on estuarine fishing grounds. While this seasonally occurs, fishers perceived the reduction in catch as being exacerbated by the negative effects of water pollution. “There is hardly anything to catch now. It rains frequently. Prior to the rainy period, I could gather as much as 100 kilos of crabs. Now I can’t even make 30 kilos … Our livelihoods are disappearing. There are less king crabs and mud crabs. Before, one could catch many shrimps and crabs. Not anymore… The rivers have to be clean again so our livelihoods can come back.” (Fisher, Paombong). In addition, illegal fishing activities were viewed as contributing to the depletion of fish stocks. “Here, there is no off-fishing season. Juveniles are caught even if those are smaller than the sizes prescribed by the government. Crabs with young are caught and sold. They remove the small crabs so that no one can tell.” (Fisher, Paombong). The selling of the illegally caught fish is not regulated in fish markets. Fishers coped with the low catch by increasing the types of gears they use for fishing. Oyster farming, which is an important alternative income source for fishers, was also disrupted with many oysters left in the farms during the lockdown.

Those who were involved in patrolling fishing grounds (*Bantay Dagat* or sea guards) reported a continuation of illegal fishing activities during the lockdown, particularly the encroachment of prohibited commercial fishing vessel into municipal waters.^5^ This was left unregulated in the early part of the lockdown when patrol activities were also stopped. In the latter part of the lockdown, fish patrols resumed their operations and managed to apprehend a number of illegal fishers. “For us, it’s about being able to help other fishers in relation to how their fishing livelihoods are affected. We continue to patrol the fishing grounds at this time, so that when things go back to normal, fishers have something they can go back to; so that they would know they can earn well enough. This is why we continue to guard the seas. That is the one thing we are capable of doing that can help.” (Fisher, Malolos).

Despite the easing of lockdown restrictions, some fishers continued to reduce their fishing activities not only because of the southwest monsoon but also because of difficulties in accessing output markets and the resultant difficulties faced in regaining costs from fishing trips (e.g. fuel).

### Market disruptions

Impacts caused by disruptions in market processes were most commonly mentioned and most emphasised by both fish farmers and fishers. For fish farmers who produced prawn, a high-value commodity for export, reduction in export led to a decrease in prawn price by as much as 50%. “Prices dropped because there was no export. Prawn sold for PhP 500/kg from PhP 900-PhP 1000/kg previously.” (Fish farmer, Paombong). The price of farmed milkfish was not significantly reduced due to continuing demand from local markets and surrounding areas since it is commonly consumed by locals. Market remained strong particularly in Metro Manila, assisted by online purchase and delivery systems and the emergence of weekly mobile markets in more affluent areas. However, there were differentiated capacities for taking advantage of this sustained demand. Large-scale fish farmers were more likely to have links with resellers in urban areas. They tended to have their own vehicles for transporting goods and were more likely to have the necessary permits and mobility passes required for transporting fish.

The prices for various types of fish from capture fisheries did not decline as much as high-value aquaculture goods because they were regularly consumed by local residents. Fishers who were interviewed reported that in some instances, a reduction in fishing trips during the lockdown and consequent decline in fish supplied in markets led to an increase in price of some caught fish when sold in fish markets. However, fishers also reported being particularly affected by changes in the timing and rules around market operations. Shortened fish market hours (5:00–11:00 am) resulted in fishers needing to bring and sell their catch to consignacions within a limited time period. Activities that used to run the whole day had to be squeezed into the morning, leading to long queues of fishers (and fish farmers) waiting for their turn. Due to physical distancing requirements inside market spaces, the number of fishers and fish farmers transacting was also limited. “Panasahan (a large fish market) was closed. It was still closed in June. During the lockdown, most goods were sold in the municipality. When the fish market opened, the number of people inside was limited. Each person could only spend around 20 minutes in the market. After that, the person would need to go out and queue up again.” (Government staff, Hagonoy). This increased the time that fisherfolks^6^ needed to spend before they could sell the fish. It also reduced the probability of their fish being bought. Moreover, the designated market hours did not fit with the daily schedule of some of the fishers who used to catch and bring their fish to the markets in the afternoon. Some fishers who caught crabs kept the live crabs in their homes and sold them the following day. Apart from limited daily market hours, market operations were further reduced from 7 to 6 days in a week to give time for disinfection. Due to these disruptions, fish producers resorted to alternative market arrangements such as peddling fish in their communities and selling in local territorial markets (called talipapa).

Aquaculture and capture fisheries were both affected by a decline in demand from restaurants and hotels. Designated buying hours and buying days and physical distancing requirements for consumers also meant less foot traffic in markets. According to interviewees, the effect of less people in markets was further compounded by widespread job losses in other sectors and reduced household income which negatively affected people’s capacities to purchase fish. “People lost work and have less money to buy fish.” (Fisher, Paombong).

### Coping strategies

The pandemic and its subsequent lockdown measures led to the emergence of numerous coping strategies such as online buying, peddling caught fish in communities, diversifying income, and growing of their own food. Online buying and selling have been practised in different parts of the Philippines, including the study area, prior to the Covid-19 crisis. This was influenced by high levels of Facebook usage even in rural areas, through free Facebook access provided for subscribers of the Philippine’s two largest telecommunication networks (i.e. Smart and Globe). With limited physical market access and restricted mobility, online selling and buying became a vital avenue for market exchange. Perishable commodities such as fish found their way into these online spaces. Fish farmers who harvested shared information about their fish in such platforms. Within small communities where mobility was not hampered by border crossings and check points, orders were increasingly made online, and goods were delivered to buyers’ homes. “The fish organisation I am a member of started to post goods online. I observed that a lot of things are now done online, even small barbecued food are ordered online and many order from it… They only sell to those who are near and can be reached by bicycle. People are afraid of going out. We don’t know who has it (Covid-19).” (Fisher, Paombong).

Apart from online marketing, fishery-dependent households also coped with market disruptions by regularly peddling caught fish within their communities. Typically, peddling is done by wives of fishers. Peddled fish is sold for lower prices than in fish markets, but this enabled households to still earn from fish catch in the face of limited access to output markets. “Even if the price of fish is low when peddled, the fish still gets sold and the peddlers don’t need to go far.” (Fisher, Paombong). It also enabled physical and economic access to fish among community members when mobility was restricted and incomes from various types of livelihoods were reduced.

Households with diversified livelihoods tended to cope better. Diversification involves having other income sources, for instance, through having other household members who are still employed in their respective jobs, holding a job in government while also earning from capture fisheries through fish traps, fishers working as labourers in nursery operations when they are not fishing, other household members earning through small-scale and informal businesses such as home-cooked and delivery of meals, and fish farmers venturing into production of other goods such as eggs. However, there were household members earning salaries from formal employment (e.g. as workers in malls and other businesses) who either lost their jobs or are working under reduced hours and reduced pay. “The impact of the Covid-19 crisis is big. There were people who were unable to get to work. Others lost their jobs. My child could not go to work because there was no public transport so she lost her job.” (Fisher, Paombong). This limited contribution to household income and the household’s capacity to cope. Reduction in number, capacity, and increase in cost of public transport also posed difficulties to people who either are going to their jobs or are looking for new employment. This limited options for coping, in favour of livelihoods that can be done in people’s immediate communities.

Growing their own food through vegetable gardens rose in importance and cushioned the negative effects of reduced purchasing power and less physical access to markets. During the lockdown, agriculture offices in municipalities distributed vegetable seeds in order to support and further encourage home gardening. Households that grew their own food in their own gardens or had direct access to fish through capture fishing reported being able to get by. For some fisheries-dependent households, having relatives who are well-off had been helpful for coping through the establishment of mutually beneficial agreements in which relatives buy the fish catch regularly.

### Social support

Fish farmers and fishers identified food aid, financial assistance, and institutional livelihood support as three types of social support that were crucial during the lockdown. Food aid in the form of relief goods were mobilised by both public and private actors throughout the country. In the study area, this was done by municipal local government units and was channelled through barangay chairpersons^7^ who implemented distribution campaigns. Relief goods were not necessarily nutritious food but were perceived as vital for averting hunger. “Support from the national and provincial government were important for avoiding hunger. Local governments and barangays also gave support especially food – rice, canned sardines, and noodles.” (Fish farmer, Paombong). Moreover, the practice of neighbours giving food to neighbours was observed. *“Here in our community, people won’t leave you to fend for yourself. Even if you don’t say anything, if they find out you are in need, they will come through for you.”* (Fisher, Paombong). Even before the pandemic, it was customary for both fish farmers and fishers to give fresh fish to their neighbours.

Financial assistance was provided by the Philippine national government through the Social Amelioration Program (SAP) implemented through the Department of Social Welfare and Development. SAP is a P200 billion (3.4€ billion) emergency subsidy programme for households that qualify as poor or belonging to the informal sector who were affected by the pandemic through the loss of livelihood. Qualified households received a one-time cash assistance of P5000–P8000 (86€–138€) for needs such as food, medicines, and toiletries. The actual assistance received differed based on one’s place of residence, with those living in areas with high living costs receiving relatively higher assistance. The distribution, however, was met with various criticisms due to problematic assessments. “There were those who were better off but received support, and those who were worse off but did not receive support.” (Fisher, Paombong). A number of other financial programmes are being planned including those that are targeted to fishers. Yet, the majority of those who were interviewed had not yet received financial assistance and were unsure whether and when they will receive it.

For fish farmers and fishers, assistance from the Bureau of Fisheries and Aquatic Resources and local government units in various aspects of their livelihoods including securing passes for mobility and connecting fisherfolks with new markets was perceived to be highly helpful. This was particularly beneficial for recognised fisherfolk organisations which were officially registered, and had established connections with government actors. Being formally recognised and linked facilitated access to required documentation such as vendors’ passes which were provided only to a limited number of people. Assistance from government actors also helped in linking members of fisherfolk organisations with markets outside of their municipality. Various fisherfolk organisations were invited to sell in mobile markets organised in various places by the Bureau of Fisheries and Aquatic Resources. One fisherfolk organisation developed agreements with a city government in Metro Manila to sell in the city’s market. This organisation consists of a mix of fish farmers and fishers who started to organise as a cooperative prior to the Covid-19 crisis and are venturing into entrepreneurial activities by selling their own or the members’ fish or by buying from fish markets and reselling elsewhere. However, a few of the trips were less than successful, resulting in having to bring back the fish that they brought. Group members divided the unsold fish among themselves and bought those for their own consumption as a matter of solidarity to prevent losses. Belonging to an organisation also enabled fisherfolks to benefit from resources of other members such as having access to a private vehicle for transporting fish. Moreover, fisherfolk organisations that have funds from regular member contributions accumulated over time used these to provide relief assistance to other members through the distribution of food. One organisation’s funds were also used as a counterpart to government assistance to finance a new project to operate an aerated tank to sell live tilapia in markets farther away. A group member viewed the pandemic as having accelerated organised and collective efforts to seek out alternative market routes.

## Discussion

Many of the impacts on aquaculture and capture fisheries presented here agree with impacts documented in other parts of the world. For instance, negative impacts in aquaculture caused by difficulties in accessing inputs and by disrupted export were widely observed in other aquaculture-producing countries in Asia (Amjath-Babu et al. 2020; Sunny et al. 2020). The importance of local food systems in maintaining food supply including the crucial role of capture fisheries has also been documented (Love et al. 2020). Practices such as the rise of online selling, buying, and delivery (Chang and Meyerhoefer 2020); home gardening for food (Steenbergen et al. 2020); the importance of production diversification (Heck et al. 2020; Savary et al. 2020); and the need for social support and safety nets (Cable et al. 2020) have been similarly discussed elsewhere. Thus, our study provides empirical contribution to globally observable patterns of pandemic impacts and emerging ways of coping in the context of aquatic food production.

In this section, we substantiate the contribution of this study beyond its value as empirical evidence. We do this in two ways. First, we focus on urgent, actionable points that actors in the study area, and similar settings can consider (sensu Flyvbjerg 2012). Second, we discuss how the findings contribute to advancing collective thinking on resilience in food systems. In particular, we unpack how local food systems linked with regional systems contribute to resilience as well as the ways they may be limited and discuss lessons on governing responses to and planning for systemic shocks.

### Actionable points for policymakers and practitioners

Food systems with more diverse food sources and livelihoods tend to be more resilient because of their modular and redundant features (Nyström et al. 2019). The co-existence of aquaculture and capture fisheries and the differentiated ways in which they were impacted by the pandemic contribute to this beneficial diversity. However, this diversity is presently under threat due to multiple factors (e.g. climate change, water pollution) and principally from land use change and infrastructure development. In particular, the recently approved construction of an international airport complex in the province of Bulacan, Central Luzon (Philippines Public-Private Partnership Center, accessed 10 August 2020), close to the study area is projected to result in a loss of about 2500 ha of fishing grounds and aquaculture areas (Environmental Justice Atlas 2018). The expected loss of livelihoods and reduction in local food production will compromise resilience of surrounding coastal social-ecological systems, with potential spill-over effects to nearby Manila and other cities which benefit from aquatic food production in Central Luzon. In view of the area’s contribution to food production, and the numerous families that depend on aquaculture and fisheries for their livelihoods who are already facing the strain of the pandemic, approval of the airport complex needs to be reconsidered and local voices from affected communities need to be included in the decision-making process.

Strong local food systems have been critical to maintaining food supply and access particularly during the highly restrictive full lockdowns (Love et al. 2020; Huizar et al. 2020; IPES-Food 2020). In the case study, both smallholder aquaculture and capture fisheries are fundamentally important to such food systems. The pandemic experience offers lessons for how to better prepare smallholder aquaculture from similar future shocks. One important strategy would be to develop fry production at the region and country levels to lessen currently high dependence on imported fry. The production and use of locally available feeds (e.g. algae, other aquatic plants) which are already available in the study area can be further strengthened by supporting local feed producers to mobilise and collectively formulate an action plan for similarly disruptive scenarios. In view of the advantages of local input sourcing, France, for instance, accelerated its plans to relocalise feed production as an important part of the government’s response to support food producers (IPES-Food 2020). In addition, strengthening the resilience of coastal social-ecological systems and aquatic food production requires that co-existent aquaculture and capture fisheries are valued for their contributions and that one sector is not compromised for the other as is the case when aquaculture development is pushed to the detriment of fisheries-dependent communities.

The International Panel of Experts on Sustainable Food Systems (2020) noted that food system vulnerability to climate and disease disruptions has been evident long before Covid-19. These vulnerabilities are shaped by political, social, economic, environmental, and climatic conditions (Bennett et al. 2020). Because primary food production sectors such as aquaculture and fisheries directly depend on natural environments for their functioning, enabling recovery and pandemic-proofing food systems requires clean and healthy environments (Savary et al. 2020). Both aquaculture and fisheries can recover faster under conditions of healthy environments that prevent incidence of fish kills, low harvest, and low catch evidently associated with degraded coastal environments. In line with this, reversing water pollution in the area is a strategic imperative. Existing institutions need contingency plans to avoid suspension of activities for environmental protection during strict lockdowns.

Informal market arrangements such as territorial markets, peddling, and open markets (Devereux et al. 2020) were observed to provide valuable alternative market arrangements for both producers and consumers because they can function under conditions of restricted mobility in small areas. Wegerif (2020) called for a need to value this sector and to improve support, for instance, by challenging the concept of ‘informal’, by ensuring better access to space, and by putting in place social safety nets for workers in this sector. However, the significantly lower price of produce sold through these channels which are desirable for consumers but not for producers requires attention. So far, government actors in the study area have provided market assistance by linking fish producers with markets outside their locality and by providing help with mobility through issuance of passes. This support may be further strengthened by expanding assistance to a larger number of fish farmers and fishers, by selecting areas for mobile markets that are more accessible to both producers and buyers, and by creating supportive measures for when goods are not sold out. For instance, the government may connect food producers with those working on distributing food aid. To address differentiated capacities between smallholder and large-scale producers to take advantage of emerging market opportunities at the region or country level, government may need to step in to provide needs-based assistance or to organise smallholder producers for cooperation. Other important points for action include more accurate identification of those in need of financial aid in the general population and timelier implementation of low-interest loan programmes for fish farmers and fishers.

Given the centrality of mobility restrictions in generating the impacts documented here, there is a need to rethink the current municipality-structured infection containment measures. While movement was still possible between municipalities particularly for essential services such as those related to food production and marketing, fragmented municipality-based approaches prevented many smallholders from carrying out essential livelihood-related activities. The establishment of safe aquaculture and fisheries production and marketing zones coordinated at the provincial level may be more effective for expediting movement of food and food producers while enforcing safety regulations.

### Lessons for resilience

While aquatic food production in the case study faced disruptions and impacts, it also exhibited a level of adaptability and resilience (see Love et al. 2020 for related findings). Here, we unpack the sources of resilience in the local food system, its limitations, and distil lessons for governing coastal social-ecological systems for responding to systemic shock.

In line with Love et al. (2020) and Brüntrup (2020), this study exemplifies the vital role of diversity, connectivity, community cooperation, and local systems (Dombroski et al. 2020; Worstell and Green 2017) in mitigating undesirable outcomes from the pandemic and contributing to resilient social-ecological systems. Debates around food systems including questions of scale and their contributions to resilience have been around for a long time. Scholars have cautioned concerning increased vulnerabilities in large-scale and highly efficient food systems and the need to maintain resilient food systems (Schipanski et al. 2016). Our case study exhibited a food system that is strongly local and is also strongly linked with larger, external markets (e.g. exports, regional markets).

Diversity in the study’s local food system comes from what one might call the peripheries. Peripheral practices (e.g. peddling) are typically less profitable than more established and central practices and are often done by those who are poor and cannot afford the costs of more profitable activities. They are also typically invisible to state planning and support. But it is precisely these characteristics that enabled peripheral practices to persist and to rapidly adapt when more central practices were disrupted. This raises questions around how the central-peripheral structure in local food systems, such as the case discussed here, can transform towards ecologies of diverse practices that do not only ensure resilience during periods of extreme disturbance but also ensure equitable distribution of benefits during periods of relative stability.

Rapid adaptability in the food system rests on a number of other factors including the proximity of aquatic food producers and consumers. Proximity enabled a faster flow of information, signals, and responses. Consequently, market bottlenecks were quickly perceived and alternative channels adopted (Dombroski et al. 2020).

However, local food systems are not a panacea. The sustained availability and accessibility of aquatic food were accompanied by differentiated reduction in the incomes of both fish farmers and fishers, similar to the observations of Eriksson et al. (2020). Importantly, local food systems are open systems that are externally linked for inputs and outputs (Love et al. 2020). In terms of inputs, while local feed availability for aquaculture is beneficial in terms of resilience, the nature of certain production systems that use both locally produced feed and industrial formulated feed means that local food systems are likely to remain embedded in broader systems beyond the local scale. In relation to outputs, the local food system’s linkage to export markets was a source of vulnerability to price drop, and income loss and its linkage to regional markets provided some buffer from export disruption. These indicate that heterogeneity in linkages serve different functions and are desirable (Love et al. 2020). Export linkages are a source of vulnerability, but they provide higher profit for certain actors. Regional linkages which are more accessible to smallholder producers contribute to resilience by enabling a redistribution of trade when the usual transactions are disrupted (Dombroski et al. 2020). In sum, local food systems embedded in broader fields are resilient when at the local scale, rearrangements can be rapidly done to meet local food needs; and at a broader scale, heterogeneous links enables a rechannel of input and output flows so that reduction in the incomes of food producers is cushioned.

In addition, the pandemic revealed the capacities of state to rapidly and comprehensively respond to a health threat and the critically important role of governance structures in shaping this response. Devolved local governance which places infection control in the hands of local government unit appears to be effective based on the relatively stabilised infection levels in the country. At the same time, an approach to infection containment at the level of municipalities results in disruptions of processes in food systems. This indicates a need to adopt a multi-level governance approach in pandemic response and recovery that combines the efficacy of local level of infection containment and higher scale food system coordination.

The ability of fish farmers and fishers to organise, mobilise, and adapt in the face of severe disruptions was an important source of resilience. This collective effort underlies the various coping strategies and channelling of support earlier described. Rapid community mobilisation was possible because of long-established relationships and collaborative experience not only between fish farmers and fishers but also with state actors. The state’s role in contributing to resilience therefore rests in its ability to exercise control to stop or limit the spread of the pandemic, while also playing a more supportive role that creates enabling conditions for communities to adapt, experiment, innovate, maintain diversity and openness in their responses, and learn.

## Conclusion

The co-existence of smallholder aquaculture and capture fisheries contributes to resilience of coastal social-ecological systems from systemic shocks such as the Covid-19 pandemic. It does so by contributing to the diversity of impact pathways, coping strategies, and social support particularly the collective efforts between fish farmers and fishers. The study provides a unique empirical contribution to the growing literature on the globally unfolding impacts of the pandemic on food systems particularly aquatic food systems and on emerging new practices that may continue after the pandemic. Based on the findings, we provide a clear set of actionable points that policymakers and practitioners can pick up. Finally, we contribute to collective thinking around the resilience of food systems particularly in the context of the pandemic by discussing sources of resilience in the local food system, highlighting its limitations and the important role of heterogeneous linkages that connect local food systems externally, and the importance of the state’s dual roles related to control and support to communities that are indispensable to resilience.

## Data Availability

Not applicable.

## Footnotes

1 For brevity, here we use the term fish to refer to the range of aquatic goods from aquaculture and capture fisheries, including crustaceans and the like, and excluding aquatic plants.

2 We assume that this applies to small-scale (municipal) fishers. A figure for large-scale (commercial) fishers was not indicated in the report.

3 Consignacions are registered businesses that function as consolidators of fish. They buy fish from fish farmers and fishers at 8–9% lower than the actual value, and sell the fish to vendors, traders, and exporters based on bidding and pay-later mechanisms.

4 Order of lockdown status from the most restrictive to the least restrictive: enhanced community quarantine (ECQ); modified enhanced community quarantine (MECQ); general community quarantine (GCQ); and modified general community quarantine (MGCQ)

5 Commercial fishing vessels are only legally permitted to fish outside municipal waters, that is, 15 km from the shore.

6 Here, the term ‘fisherfolks’ is used to refer collectively to fish farmers and fishers.

7 A barangay is the smallest administrative unit in the Philippines, and it is headed by a barangay chairperson who is elected for 3 years.
